# Supplementary material for: Factors predicting students’ performance in the final pediatrics OSCE
Source: PLoS One. 2020 Sep 2;15(9):e0236484. doi: 10.1371/journal.pone.0236484 (PMC7467284; doi:10.1371/journal.pone.0236484)
Supplement: S1 Dataset — (DOCX) [file pone.0236484.s001.docx]

| **Descriptives** | | | | | | | | | |
| --- | --- | --- | --- | --- | --- | --- | --- | --- | --- |
|  | | N | Mean | Std. Deviation | Std. Error | 95% Confidence Interval for Mean | | Minimum | Maximum |
|  |  |  |  |  |  | Lower Bound | Upper Bound |  |  |
| End of block OSCE year 2013-2016 | 1 | 96 | 79.3854 | 6.37573 | .65072 | 78.0936 | 80.6773 | 66.00 | 95.50 |
|  | 2 | 94 | 78.3617 | 6.89881 | .71156 | 76.9487 | 79.7747 | 59.00 | 93.50 |
|  | 3 | 96 | 80.1771 | 5.94004 | .60625 | 78.9735 | 81.3806 | 59.00 | 92.50 |
|  | Total | 286 | 79.3147 | 6.43379 | .38044 | 78.5659 | 80.0635 | 59.00 | 95.50 |
| Final OSCE year 2013-2016 | 1 | 96 | 77.4740 | 6.34465 | .64755 | 76.1884 | 78.7595 | 61.00 | 92.00 |
|  | 2 | 94 | 77.1862 | 6.48902 | .66929 | 75.8571 | 78.5153 | 59.00 | 94.00 |
|  | 3 | 96 | 79.1719 | 5.83629 | .59566 | 77.9893 | 80.3544 | 64.00 | 92.00 |
|  | Total | 286 | 77.9493 | 6.26772 | .37062 | 77.2198 | 78.6788 | 59.00 | 94.00 |

| **ANOVA** | | | | | | |
| --- | --- | --- | --- | --- | --- | --- |
|  | | Sum of Squares | df | Mean Square | F | Sig. |
| End of block OSCE year 2013-2016 | Between Groups | 157.247 | 2 | 78.624 | 1.912 | .150 |
|  | Within Groups | 11639.931 | 283 | 41.130 |  |  |
|  | Total | 11797.178 | 285 |  |  |  |
| Final OSCE year 2013-2016 | Between Groups | 219.924 | 2 | 109.962 | 2.835 | .060 |
|  | Within Groups | 10976.091 | 283 | 38.785 |  |  |
|  | Total | 11196.015 | 285 |  |  |  |

| **Multiple Comparisons** | | | | | | | |
| --- | --- | --- | --- | --- | --- | --- | --- |
| LSD | | | | | | | |
| Dependent Variable | (I) Rotations 1, 2 & 3 | (J) Rotations 1, 2 & 3 | Mean Difference (I-J) | Std. Error | Sig. | 95% Confidence Interval | |
|  |  |  |  |  |  | Lower Bound | Upper Bound |
| End of block OSCE year 2013-2016 | 1 | 2 | 1.02371 | .93059 | .272 | -.8080 | 2.8555 |
|  |  | 3 | -.79167 | .92568 | .393 | -2.6138 | 1.0304 |
|  | 2 | 1 | -1.02371 | .93059 | .272 | -2.8555 | .8080 |
|  |  | 3 | -1.81538 | .93059 | .052 | -3.6471 | .0164 |
|  | 3 | 1 | .79167 | .92568 | .393 | -1.0304 | 2.6138 |
|  |  | 2 | 1.81538 | .93059 | .052 | -.0164 | 3.6471 |
| Final OSCE year 2013-2016 | 1 | 2 | .28779 | .90367 | .750 | -1.4910 | 2.0665 |
|  |  | 3 | -1.69792 | .89890 | .060 | -3.4673 | .0715 |
|  | 2 | 1 | -.28779 | .90367 | .750 | -2.0665 | 1.4910 |
|  |  | 3 | -1.98570^*^ | .90367 | .029 | -3.7645 | -.2069 |
|  | 3 | 1 | 1.69792 | .89890 | .060 | -.0715 | 3.4673 |
|  |  | 2 | 1.98570^*^ | .90367 | .029 | .2069 | 3.7645 |
| *. The mean difference is significant at the 0.05 level. | | | | | | | |

| **rotation 1** | | | | | |
| --- | --- | --- | --- | --- | --- |
|  | N | Minimum | Maximum | Mean | Std. Deviation |
| End of block OSCE year 2013-2016 | 96 | 66.00 | 95.50 | 79.3854 | 6.37573 |
| Final OSCE year 2013-2016 | 96 | 61.00 | 92.00 | 77.4740 | 6.34465 |
| Written final | 96 | 50.00 | 91.00 | 74.7406 | 7.32396 |
| Valid N (listwise) | 96 |  |  |  |  |

| **Rotation 2** | | | | | |
| --- | --- | --- | --- | --- | --- |
|  | N | Minimum | Maximum | Mean | Std. Deviation |
| End of block OSCE year 2013-2016 | 94 | 59.00 | 93.50 | 78.3617 | 6.89881 |
| Final OSCE year 2013-2016 | 94 | 59.00 | 94.00 | 77.1862 | 6.48902 |
| Written final | 94 | 55.30 | 89.00 | 74.7862 | 6.86172 |
| Valid N (listwise) | 94 |  |  |  |  |

| **Rotation 3** | | | | | |
| --- | --- | --- | --- | --- | --- |
|  | N | Minimum | Maximum | Mean | Std. Deviation |
| End of block OSCE year 2013-2016 | 96 | 59.00 | 92.50 | 80.1771 | 5.94004 |
| Final OSCE year 2013-2016 | 96 | 64.00 | 92.00 | 79.1719 | 5.83629 |
| Written final | 96 | 57.70 | 91.00 | 75.2396 | 6.72522 |
| Valid N (listwise) | 96 |  |  |  |  |

| **All 1, 2 & 3 ROTATIONS** | | | | | |
| --- | --- | --- | --- | --- | --- |
|  | N | Minimum | Maximum | Mean | Std. Deviation |
| End of block OSCE year 2013-2016 | 286 | 59.00 | 95.50 | 79.3147 | 6.43379 |
| Final OSCE year 2013-2016 | 286 | 59.00 | 94.00 | 77.9493 | 6.26772 |
| Written final | 286 | 50.00 | 91.00 | 74.9231 | 6.95495 |
| Valid N (listwise) | 286 |  |  |  |  |

**EOBOSCE vs Final OSCE**

| **Groups ( End Block OSCE vs Final OSCE)** | **Mean & SD** | **P value** |
| --- | --- | --- |
| Rotation 1 (yrs 2013-16 Total 96 ) vs Final (yrs 2013-16 Total 96) | 79.39±6.38 vs 77.47±6.34 | 0.038* |
| Rotation 2 (yrs 2013-16 T=94) vs Final (yrs 2013-16 T=94) | 78.36±6.90 vs 77.19±6.48 | 0.23 |
| Rotation 3 (yrs 2013-16 T=96) vs Final (yrs 2013-16 T=96) | 80.18±5.94 vs 79.17±5.84 | 0.24 |
| All rotations 1+2+3 (yrs 2013-16 T=286) vs Final 1+2+3 (yrs 2013-16 T=286) | 79.31±6.43 vs 77.95 ±6.27 | 0.01* |

**Final Written vs Final OSCE**

| **Rotation Groups (Final Written vs Final OSCE)** | **Mean & SD** | **P value** |
| --- | --- | --- |
| Rotation 1 (yrs 2013-16 Total 96 ) vs Final (yrs 2013-16 Total 96) | 74.74±7.32 vs 77.47±6.34 | 0.01* |
| Rotation 2 (yrs 2013-16 T=94) vs Final (yrs 2013-16 T=94) | 74.78±6.86 vs 77.19±6.48 | 0.01* |
| Rotation 3 (yrs 2013-16 T=96) vs Final (yrs 2013-16 T=96) | 75.24±6.73 vs 79.17±5.84 | <0.0001* |
| All rotations 1+2+3 (yrs 2013-16 T=286) vs Final 1+2+3 (yrs 2013-16 T=286) | 74.92±6.95 vs 77.95 ±6.27 | <0.0001* |

**Within GROUPS ( END OF BLOCK OSCE)**

| **Rotation with in groups of OSCE** | **Mean & SD** | **P value** |
| --- | --- | --- |
| Rotation **1** (yrs 2013-16 Total 96 ) **vs** Rotation **2** (yrs 2013-16 Total 94) | 79.39±6.38 vs 78.36±6.90 | 0.29 |
| Rotation **1** (yrs 2013-16 T=96) **vs**  **3** (yrs 2013-16 T=96) | 79.39±6.38 vs 80.18 ±5.94 | 0.38 |
| Rotation **2** (yrs 2013-16 T=96) **vs**  **3** (yrs 2013-16 T=96) | 78.36±6.90 vs 80.18 ±5.94 | 0.052* |

**Within Groups Final OSCE**

| **Rotation with in groups** | **Mean & SD** | **P value** |
| --- | --- | --- |
| Rotation 1 (yrs 2013-16 Total 96 ) vs Rotation 2 (yrs 2013-16 Total 94) | 77.47±6.34 vs 77.19±6.49 | 0.76 |
| Rotation 1 (yrs 2013-16 T=96) vs 3 (yrs 2013-16 T=96) | 77.47±6.34 vs 79.17±5.84 | 0.06 |
| Rotation 2 (yrs 2013-16 T=96) vs 3 (yrs 2013-16 T=96) | 77.19±6.48 vs 79.17±5.84 | 0.028* |

| **Rotations 1, 2 & 3 * Grades Crosstabulation** | | | | | | |
| --- | --- | --- | --- | --- | --- | --- |
| Count | | | | | | |
|  | | Grades | | | | Total |
|  |  | A | B | C | F |  |
| Rotations 1, 2 & 3 | 1 | 3 | 25 | 66 | 2 | 96 |
|  | 2 | 4 | 18 | 70 | 2 | 94 |
|  | 3 | 2 | 30 | 61 | 3 | 96 |
| Total | | 9 | 73 | 197 | 7 | 286 |

| **Grades** | | | | | |
| --- | --- | --- | --- | --- | --- |
|  | | Frequency | Percent | Valid Percent | Cumulative Percent |
| Valid | A | 9 | 3.1 | 3.1 | 3.1 |
|  | B | 73 | 25.5 | 25.5 | 28.7 |
|  | C | 197 | 68.9 | 68.9 | 97.6 |
|  | F | 7 | 2.4 | 2.4 | 100.0 |
|  | Total | 286 | 100.0 | 100.0 |  |

| **All groups year 2013-16** | | | | | |
| --- | --- | --- | --- | --- | --- |
|  | N | Minimum | Maximum | Mean | Std. Deviation |
| End of Block written | 286 | 40.30 | 85.00 | 67.6622 | 8.15429 |
| End of block OSCE | 286 | 59.00 | 95.50 | 79.3147 | 6.43379 |
| Written final | 286 | 50.00 | 91.00 | 74.9231 | 6.95495 |
| Final OSCE | 286 | 59.00 | 94.00 | 77.9493 | 6.26772 |
| Valid N (listwise) | 286 |  |  |  |  |
